# A Catchment and Location-Allocation Analysis of Mammography Access in Delaware, US: Implications for disparities in geographic access to breast cancer screening

**DOI:** 10.21203/rs.3.rs-2600236/v1

**Published:** 2023-02-28

**Authors:** Jessica L. Webster, Neal D. Goldstein, Jennifer R. Rowland, Catherine M. Tuite, Scott D. Siegel

**Affiliations:** Drexel University Dornsife School of Public Health; Drexel University Dornsife School of Public Health; Christiana Care Health System; Christiana Care Health System; Christiana Care Health System

**Keywords:** breast cancer, mammography, disparity, catchment, location-allocation

## Abstract

**Background:**

Despite a 40% reduction in breast cancer mortality over the last 30 years, not all groups have benefited equally from these gains. A consistent link between later stage of diagnosis and disparities in breast cancer mortality has been observed by race, socioeconomic status, and rurality. Therefore, ensuring equitable geographic access to screening mammography represents an important priority for reducing breast cancer disparities. This study conducted a catchment and location-allocation analysis of mammography access in Delaware, a state that is representative of the US in terms of race and urban-rural characteristics and experiences an elevated burden from breast cancer.

**Methods:**

A catchment analysis using the ArcGIS Pro Service Area analytic tool characterized the geographic distribution of mammography sites and Breast Imaging Centers of Excellence (BICOEs). Poisson regression analyses identified census tract-level correlates of access. Next, the ArcGIS Pro Location-Allocation analytic tool identified candidate locations for the placement of additional mammography sites in Delaware according to several sets of breast cancer screening guidelines.

**Results:**

The catchment analysis showed that for each standard deviation increase in the number of Black women in a census tract, there were 64% (95% CI, 0.18–0.66) fewer mammography units and 85% (95% CI, 0.04–0.48) fewer BICOEs. The more rural counties in the state accounted for 41 % of the population but only 22% of the BICOEs. The results of the location-allocation analysis depended on which set of screening guidelines were adopted, which included increasing mammography sites in communities with a greater proportion of younger Black women and in rural areas.

**Conclusions:**

The results of this study illustrate how catchment and location-allocation analytic tools can be leveraged to guide the equitable selection of new mammography facility locations as part of a larger strategy to close breast cancer disparities.

## Introduction

Breast cancer is the leading cause of cancer incidence and the second leading cause of cancer mortality among US women.^1^ Advances in early detection and treatment are largely believed to have contributed to the 40% reduction in breast cancer mortality observed over the last 30 years,^1^ but not all groups have benefited equally from these advances. Persistent breast cancer disparities have been observed by race, socioeconomic status (SES), and geographic area. Black women have a 40% higher breast cancer mortality rate relative to White women despite similar incidence rates between the racial groups.^1^ This mortality rate grows to 86% higher for younger Black vs. White women,^1^ owing to the greater risk that Black women have of being diagnosed with advanced-stage breast cancer before age 50.^2^ Other research found 14% lower breast cancer five-year survival rates for low-SES patients relative to their more advantaged peers.^3^ Approximately two-thirds of this disparity was attributable to conditions at presentation, including later stage at diagnosis. Finally, geographic characteristics including neighborhood measures of disadvantage (e.g., SES, segregation)^4–10^ and rurality^11–14^ have been associated with later stage at diagnosis and poorer breast cancer survival.

Given the consistent link between later stage at diagnosis and disparities in breast cancer outcomes across multiple population subgroups, ensuring equitable access to screening mammography represents an important goal of breast cancer prevention and early detection. Screening mammography has been a central component of breast cancer programs in the US over the last 30 + years.^15^ A review of the evidence shows that screening mammography can reduce breast cancer mortality by at least 40% when completed on an annual basis beginning at age 40.^16^ More recent studies have helped to establish that the benefits of screening are independent of treatment advances.^17,18^ Screening mammography decreases mortality by detecting tumors at a smaller size and an earlier stage, when therapy is more effective.^19^

Decisions about how to allocate mammography resources to ensure equitable geographic access are contingent on which set of screening guidelines are adopted. Multiple US medical organizations have issued screening guidelines that vary along several dimensions, including the recommended age of initiation and screening interval.^20^ On one end of the spectrum, the American College of Radiology (ACR)^21^ recommends that women of average risk for breast cancer should initiate annual screening mammography at age 40 to maximize life-years gained. The National Comprehensive Cancer Network (NCCN)^22^ and the American Society of Breast Surgeons (ASBrS)^23^ have issued similar recommendations. On the other end of the spectrum, the US Preventive Services Task Force (USPSTF) published a “B” recommendation (i.e., “moderate to substantial net benefit”) for women of average risk to initiate biennial screening mammography at age 50.^24^ The USPSTF issued a lower level “C” recommendation (i.e., “small net benefit”) for women ages 40–49, citing the need to balance the benefits of screening against the potential harms of overdiagnosis and overtreatment.^24^ Other organizations, such as the American Cancer Society (ACS), have issued recommendations that fall somewhere between the ACR and USPSTF guidelines (i.e., annual mammography initiated at age 45 before transitioning to biennial screening beginning at age 55).^25^ Ensuring equitable access to mammography facilities under the ACR relative to the USPSTF guidelines would likely require significantly greater mammography screening capacity given the earlier age of initiation and shorter screening interval, particularly in rural and other disadvantaged areas where access is typically limited.

Beyond the existing screening guidelines, increasing awareness of racial differences in the age distributions of breast cancer incidence and mortality has called for action to advance health equity. While some organizations, such as the ACR and ASBrS, have called for formal lifetime breast cancer risk assessment by the age of 30 for Ashkenazi Jewish and Black/African American women to identify those who would benefit from risk reduction strategies including earlier screening with mammography and/or MRI, some have suggested establishing race-based imaging guidelines.^26,27^ Race-based guidelines refer to screening schedules based on a patient’s race. It has been argued that the USPSTF guidelines contribute to racial disparities and should be specifically modified to recommend screening initiation at age 40 for Black women.^26^ As noted, the ACR and other organizations do currently recommend screening for all women beginning at age 40, regardless of race. However, under the Affordable Care Act, private insurers and Medicaid are only required to cover preventive services recommended by the USPSTF at the B grade or higher.^28^ Thus, the USPSTF recommendations may impact access to services. In addition, evidence has shown that the current USPSTF guidelines have led to a decrease in clinicians recommending mammography to younger Black women.^29^ The USPSTF is currently in the process of updating their guidelines and is explicitly considering the key question of whether the benefits and harms of screening differ by population characteristics, including race.^30^ Addressing this specific question, a recent simulation modeling study evaluated how the USPSTF screening mammography guidelines could be made more equitable for Black women in the US.^31^ Simulation modeling was required because Black women have been historically underrepresented in screening trials, precluding analyses stratified by race.^32^ The authors reported that initiating biennial screening for Black women beginning at age 40 would achieve the same benefits of biennial screening beginning at age 50 observed for White women, which could reduce the Black-White difference in breast cancer mortality by 57%.^31^ It should be noted that race-based approaches to medicine have been critiqued on multiple ethical and pragmatic grounds.^33–35^ Nevertheless, if the USPSTF was to utilize a race-based approach when updating their screening guidelines to address the breast cancer disparity observed for younger Black women, instead of adopting more inclusive guidelines recommended by the ACR/NCCN/ASBrS, this could have implications for the allocation of mammography resources.

In addition to allocating mammography facilities on the basis of screening guidelines, more recent evidence has pointed to the importance of mammography facility quality.^36,37^ Quality measures for mammography facilities include academic setting, mammograms being read exclusively by breast imaging specialists, and the availability of digital mammography.^36^ Designation as a Breast Imaging Center of Excellence (BICOE) by the ACR^38^ has been used in prior research to understand the link between comprehensive assessments of breast imaging facilities and racial disparities in stage at diagnosis.^37^ Importantly, community-based programs designed to equitably improve access to high-quality screening mammography facilities have been shown to meaningfully reduce disparities in breast cancer mortality.^39,40^

This study had two objectives related to evaluating and improving equitable access to screening mammography facilities in Delaware. We focused our analyses on Delaware because it is broadly representative of the US in terms of race and urban-rural characteristics^41,42^ and has among the highest incidence rates in the US for breast cancer among younger Black women^43^ and triple negative breast cancer,^44^ an aggressive subtype of breast cancer that is more likely to present at a younger age.^45^ In addition, Delaware has the cancer care infrastructure necessary to implement population-level prevention programs and a track record of eliminating other cancer disparities with improved screening programs.^46^ The first objective was to conduct a statewide catchment analysis of mammography access. The catchment analysis described the location of mammography facilities, including BICOE-accredited facilities, and evaluated whether these facilities were patterned by sociodemographic characteristics. The second objective was to conduct a location-allocation analysis to identify candidate locations for the establishment of new mammography facilities to optimize equitable access according to the existing ACR guidelines and to the USPSTF guidelines with or without race-based considerations.

## Methods

### Data sources

Census tract measures of population size; number of women aged 40–49, 50–74, and older than 74; percentage of women who are Black; area deprivation; and percentage of households with at least one vehicle were obtained from the U.S. Census Bureau’s American Community Survey 5-year estimates.^47^ Area deprivation was operationalized as a Z-score composite of education, employment, income and poverty, and household composition, where a higher score indicates greater deprivation.^48^ The per tract number of bus stops were obtained from the Delaware open data portal.^49^ Mammography facilities were compiled from two sources, the U.S. Food and Drug Administration certified facility list^50^ and the American College of Radiology’s accredited facility list,^51^ the latter resource also identifying whether a facility was a BICOE. We retrieved all sites in Delaware as well as sites within border-adjacent ZIP codes in Maryland and Pennsylvania recognizing that Delaware residents may cross state lines for mammography services. For sites in Delaware, the number of active mammography units per site was obtained from the Delaware Department of Health and Social Services. This information was used to estimate site capacity, or how many screening mammograms a site could perform each year. Using a calculation by Young and colleagues,^52^ and the definition of maximum capacity of three mammograms per mammography unit per business hour,^53^ capacity of a facility with one mammography unit was estimated at an average of 4,500 screenings per unit per year. For sites with more than one mammography unit this value was multiplied by the number of units at that site. Lastly, census tract and county boundary definition shapefiles were downloaded from the U.S. Census Bureau.^54^ Across Delaware’s three counties, we explored heterogeneity by tracts inside or outside of New Castle County (i.e., Kent and Sussex counties), as New Castle County is more urban and contains the relatively densely populated city of Wilmington, while Kent and Sussex counties tend to be more rural (see Supplemental Fig. 1).

### Statistical analysis

First, we used descriptive statistics to summarize statewide and county-specific census tract measures of population, transportation (a proxy for accessibility), and mammography sites. We then performed a catchment analysis using the ArcGIS Pro Service Area analytic tool to identify areas within 15, 30, and greater than 30 minutes driving time from each existing mammography site, with driving time serving as an indicator of geographic access. This Service Area analytic tool calculates the maximum driving distance from a point that can be traveled along a road network,^55^ representing a service catchment area.

As part of this catchment analysis, Poisson regression models predicted the independent census tract correlates (enumerated earlier) of the number of mammography facilities, units, BICOE facilities, and BICOE units statewide and separately for New Castle County and Kent and Sussex Counties. All independent variables were standardized for modeling. These ecological models included the census tract population as an offset to account for population differences and the estimates may be interpreted as relative risks per standard deviation change with corresponding 95% confidence intervals (CIs).

Next, we used the ArcGIS Pro Location-Allocation analytic tool to identify candidate locations for the placement of additional mammography sites in Delaware. This Location-Allocation tool uses heuristic procedures to identify locations for services based on location-specific demand.^56^ Within this analysis, the services are mammography screenings, and demand represents the people eligible to receive these services according to screening guidelines. Three competing specifications of demand were used in these analyses. The primary specification was based on the USPSTF recommendation of biennial mammography screenings for women aged 50 to 74 years.^24^ The second specification was based on a simulation study by Chapman and colleagues that recommends initiating biennial screening in Black women at age 40, in addition to the USPSTF’s recommendation of biennial screening for all women aged 50–74 years.^31^ The third specification was based on the ACR recommendation of annual mammograms for women age 40 and older.^21^ These three specifications of demand were calculated for each census tract and represented the number of women who would be eligible to receive screening mammography each year. Population-weighted centroids were calculated for each census tract and represented the location of the women residing in that census tract (i.e., location of demand). New mammography sites could be placed anywhere within the census tracts, with candidate locations created using the ArcGIS Pro Fishnet tool. To identify locations of new mammography sites that would fill in the gaps in demand that the current mammography sites are unable to reach, the location-allocation analysis took into consideration the locations and the capacities of the existing mammography sites. This was achieved using the Maximize Capacitated Coverage problem type, which selects candidate sites such that the maximum amount of demand is served without exceeding the capacity of the sites.^57^ Candidate sites were assumed to have a single mammography unit with the same capacity as existing facilities with one unit. For all demand specifications, the location-allocation analyses were run three times, allowing for the addition of one, three, or five new mammography sites. Driving time from demand points to mammography sites was used to determine appropriate location allocation, and a cut-off of 20-minutes driving time was specified as the maximum amount of time an individual would likely travel to a site. Finally, we used the Location-Allocation tool to identify existing sites that might benefit from a conversion to a BICOE. This analysis used the primary USPSTF demand specification and all other parameters used in the previously described location-allocation analyses, with one difference: only BICOE sites and their capacities were used as the existing locations, while all non-BICOE sites were specified as candidate locations.

All analyses were conducted in R version 3.6.3 (R Foundation for Statistical Computing, Vienna, Austria) and ArcGIS Pro version 2.9.0 (ESRI, Carrboro, NC). Computational codes in R are available to download from: https://doi.org/10.5281/zenodo.7430255

## Results

Across 214 populated census tracts (943,732 residents) in Delaware, there were 30 mammography facilities containing 44 total units, of which 9 sites (30%) were BICOE (20 total units, 67%). New Castle County (555,036 residents, 59% of population) contained 16 facilities (53% of state), 25 units (57% of state), 7 BICOE sites (78% of state), and 16 BICOE mammogram units (80% of state), while Kent and Sussex Counties (388,696 residents, 41% of population) contained 14 facilities (47% of state), 19 units (43% of state), 2 BICOE sites (22% of state), and 4 BICOE units (20% of state). There were 6 mammography facilities outside of Delaware but in bordering ZIP codes: 4 in Pennsylvania and 2 in Maryland. One of the Maryland sites was a BICOE.

Table 1 presents census tract measures of population, transportation, and mammography sites in Delaware overall and by county. On average by census tract, New Castle County had fewer women aged 50–74 years (649 versus 824) and over 74 years (155 versus 209), a higher proportion of Black women (26% versus 16%), a lower proportion of households with a vehicle (91 % versus 95%), and a greater number of bus stops (14 versus 7) compared to Kent and Sussex Counties.

### Catchment Analysis

**Figure 1:**
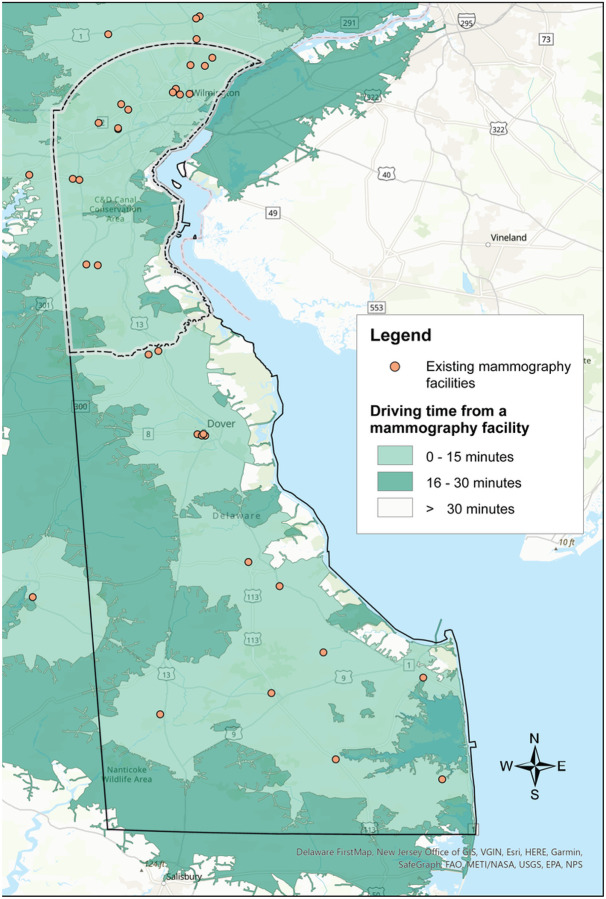
Delaware mammography facilities and average driving time from the population-weighted census tract centroid.

The majority of Delaware’s population lives in the northernmost county, New Castle County, which encompasses 130 (61 %) census tracts (Supplemental Fig. 1). Results of the service area analysis are illustrated in Fig. 1, showing a map of the existing mammography sites in Delaware, plus an additional six sites in adjacent ZIP codes (four in Pennsylvania and two in Maryland), with shaded areas depicting the driving distance from each site. In New Castle County, 98% of census tracts are within 15 minutes, and none of the census tracts over 30 minutes driving time to a mammography site. Outside of New Castle County (Kent and Sussex) almost 78% of census tracts are within 15 minutes driving time, and just over 2% of census tracts are over 30 minutes driving time from a mammography site (Supplemental Table 1).

Table 2 and Supplemental Table 2 present the results of the Poisson regression models for the presence of mammography facilities and units and BICOE facilities and units, respectively. Several findings were apparent from these models. First, as the number of women aged 40–49 increased in a given census tract, the number of mammography facilities increased 1.89 times (95% CI: 1.15, 3.24) and 3.03 times (95% CI: 1.35, 7.23) statewide and in New Castle County, respectively, and the number of units increased 2.09 times (95% CI: 1.33, 3.41) and 4.92 times (95% CI: 2.33, 11.3) statewide and in New Castle County, respectively, for each standard deviation change. This trend was also observed for the number of BICOE facilities and units in Delaware and New Castle County; BICOE model results are not available for Kent and Sussex Counties due to the small number of BICOE facilities and units outside of New Castle County. Second, there was a trend toward fewer mammography facilities and units statewide and in New Castle County as the number of women aged 50–74 increased per census tract. Third, as the percentage of Black women in the census tracts increased, there were fewer mammography facilities and units statewide and by county. For example, for each standard deviation increase in Black women in a census tract, there were 64% fewer units in Delaware (95% CI: 0.18, 0.66) and 84% fewer units in New Castle County (95% CI: 0.05, 0.49). This finding was strongest for BICOE facilities and units. For example, for each standard deviation increase in Black women in a census tract, there were 85% fewer BICOE units in Delaware (95% CI: 0.04, 0.48) and 98% fewer BICOE units in New Castle County (95% CI: 0.00, 0.18). Fourth, we also noted opposing associations for the transportation predictors, where a greater proportion of households with at least one vehicle was associated with a *decreased* rate of mammography facilities and units, and a greater number of bus stops was associated with an *increased* rate of mammography facilities and units.

### Location-allocation analysis

**Figure 2:**
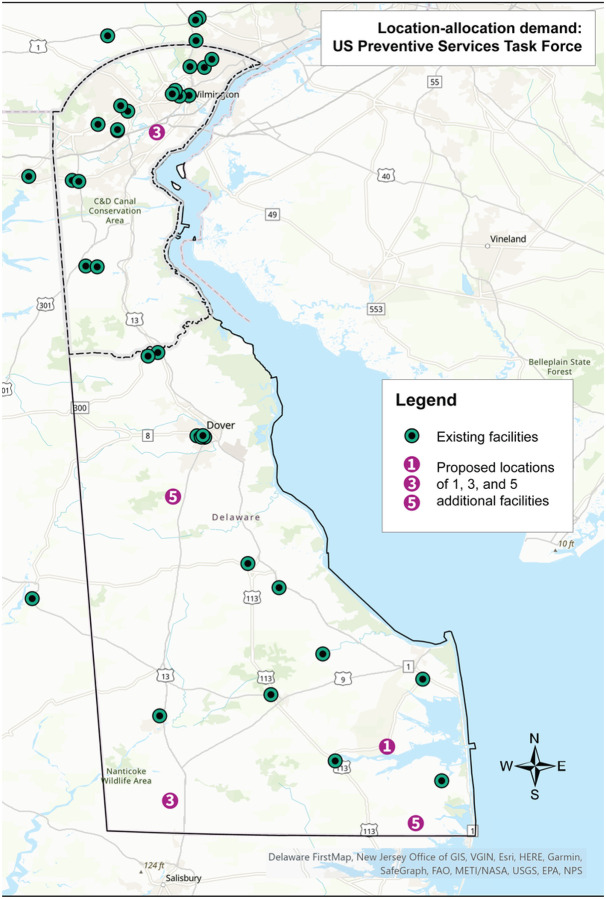
Results of the location-allocation analysis using the demand specification of all women per the U.S. Preventive Services Task Force mammography screening guideline. Existing sites in Delaware and ZIP code adjacent locations in Pennsylvania and Maryland are shown as black/green circles. The numbered circles indicate where 1, 3, and 5 additional sites should be placed based on demand. These numbers are cumulative.

**Figure 3:**
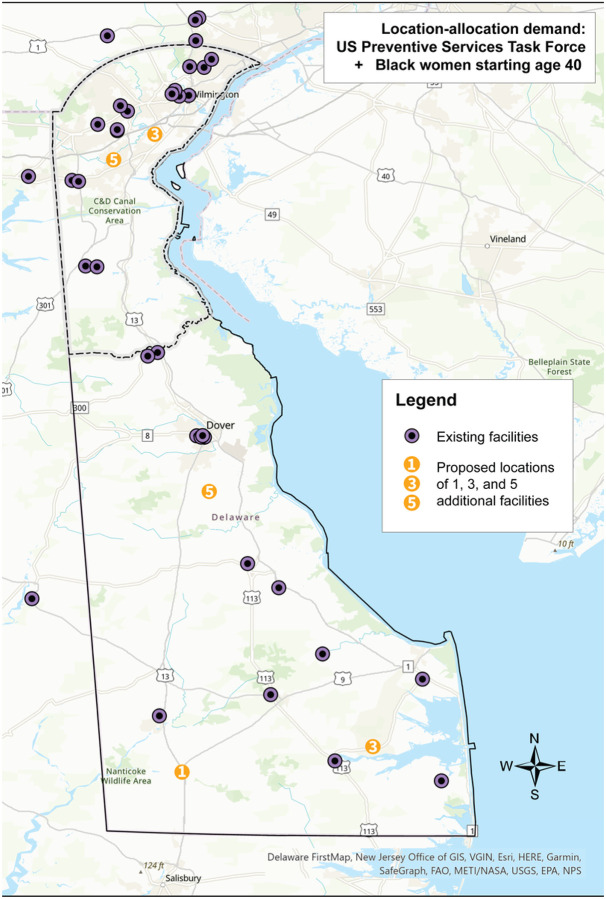
Results of the location-allocation analysis using the demand specification of all women per the U.S. Preventive Services Task Force mammography screening guideline plus biennial screening in Black women starting at age 40. Existing sites in Delaware and ZIP code adjacent locations in Pennsylvania and Maryland are shown as black/purple circles. The numbered circles indicate where 1, 3, and 5 additional sites should be placed based on demand. These numbers are cumulative.

**Figure 4:**
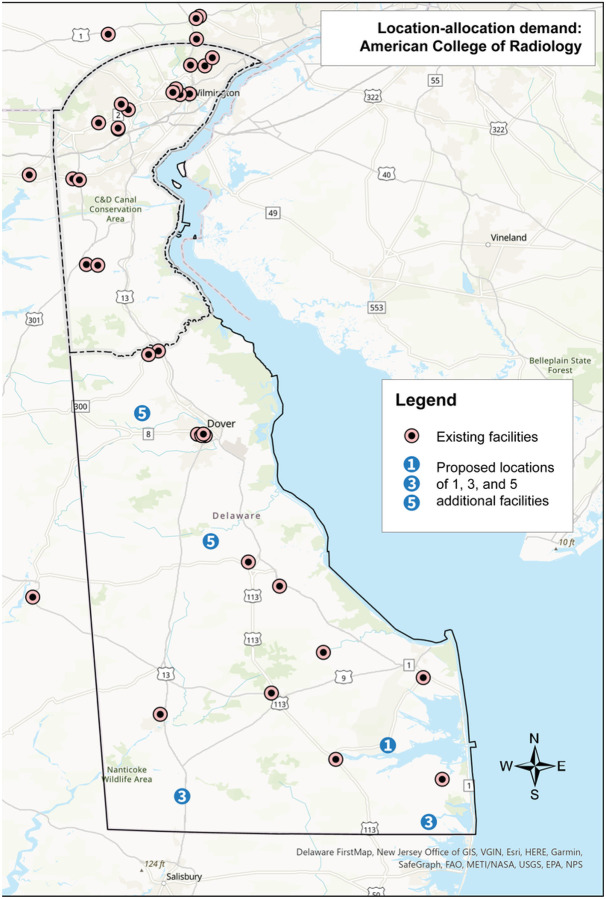
Results of the location-allocation analysis using the demand specification of all women per the American College of Radiology mammography screening guideline. Existing sites in Delaware and ZIP code adjacent locations in Pennsylvania and Maryland are shown as black/green circles. The numbered circles indicate where 1, 3, and 5 additional sites should be placed based on demand. These numbers are cumulative.

Results of the location-allocation analysis using the USPSTF demand specification, the race-based specification, and the ACR specification are depicted in Figs. 2–4, respectively. Using the USPSTF demand specification, if adding one additional mammography site to the 36 existing sites within Delaware and the adjacent ZIP codes in Pennsylvania and Maryland, the best location based on demand would be in the southeast region of the state near the town of Millsboro. If adding three new sites, which would include the Millsboro location, one additional site would be placed in the southwestern region of the state near the town of Laurel, as well as in the northern region of the state, near the city of New Castle.

And finally, when adding five new sites, which would include the three just described, one additional site would be placed in the southeastern region of the state near the town of Selbyville, and one in the center of the state near the town of Felton. Changing the demand specification to the race-based recommendations shifted the locations of the proposed mammography sites. Instead of three proposed sites in the southern region of the state, one central site, and one northern site, this analysis proposed two in the south near the towns of Laurel and Millsboro, one central near the town of Felton, and two in the north near the city of New Castle and the Bear area. Lastly, using the ACR specification, all five proposed sites fell outside of New Castle County: one west of Dover, one in the town of Milford, one in the town of Laurel, one near the town of Selbyville, and one near the town of Millsboro. Overall, the addition of five new mammography sites reduced driving time on average by 4% for the USPSTF and the race-based specifications, and by 2% for the ACR specification. New Castle County experienced the greatest improvement in driving time using the USPSTF and race-based specifications, with the addition of five new sites reducing averaged driving time by 8%. Using the ACR specification, Sussex County experienced the greatest improvement in driving time with a reduction of 12%.

Results from the BICOE location-allocation analysis using the USPSTF demand specification are depicted in Supplemental Fig. 2. Of the 10 existing BICOE sites, one is in Maryland, two are in Kent County, and seven are in New Castle County. The five existing non-BICOE sites identified by the location-allocation analysis for consideration for conversion to BICOE include one site in New Castle County (in the city of Wilmington) and four sites in Kent and Sussex Counties (in the towns of Millsboro, Rehoboth Beach, Seaford, and Milford).

## Discussion

In a catchment analysis of mammography access in Delaware, the state with among the highest rates of breast cancer among younger Black women in the US, we observed what initially appeared to be adequate access to screening. In New Castle County, the most populous county in the state, 98% of the population lived within a 15-minute drive of a mammography facility. In the other, more rural two counties in the state, 78% and 98% of the population lived within a 15-minute and 30-minute drive of a facility, respectively. Across all racial groups, we observed a positive relationship between the number of younger women (i.e., 40–49 years) and the number of mammography facilities/units and BICOEs statewide. We did not observe significant associations between the number of women currently eligible for screening mammography under the current USPSTF guidelines and measures of mammography access, with the exception of a significantly decreased number of units relative to the number of women 50–74 years in New Castle County census tracts.

When mammography access was considered through a health equity lens, we found preliminary evidence suggestive of disparities related to race and rurality. For every standard deviation increase in the number of Black women in a census tract, there were 64% fewer mammography units statewide. In New Castle County, the county with the largest Black population in the state, we observed 84% fewer units for every standard deviation increase in the number of Black women in a census tract. This finding was even stronger for BICOE units: for every standard deviation increase in the number of Black women in a census tract, there were 85% and 98% fewer BICOE units statewide and in New Castle County, respectively (with similar results observed for facilities). Fewer mammography facilities and units in predominantly Black census tracts points to a potential disparity in screening access. Regarding disparities by SES, we did not find a significant association between area deprivation and the number of mammography facilities or units in New Castle County, Kent and Sussex Counties, or statewide. Regarding disparities by rurality, the number of statewide facilities and units were proportional to the population size for New Castle County and Kent and Sussex Counties. However, while 100% of the census tracts in New Castle County were within a 30-minute drive of a mammography facility, two census tracts in the southern part of Delaware had drive times greater than 30 minutes. In addition, the more rural counties in the state accounted for 41 % of the population but only 22% of the BICOEs.

The results of the location-allocation analysis using the USPSTF demand specification highlighted the opportunity to increase access in the more rural, southern part of the state. When adding five additional mammography sites, four were proposed for the southern part of the state and one in New Castle County. When five existing non-BICOE mammography facilities were considered for conversion to BICOE sites, four were identified in the southern part of the state and one in Wilmington, the largest city in the state. This finding is consistent with other research, which has found that among the greatest disparities in the geographic access to mammography facilities exist in small towns and rural areas.^12,52,58^ When the results of these analyses are considered for the USPSTF guidelines with race-based screening demand specifications, three additional sites were proposed for the southern part of the state and two additional sites were proposed for New Castle County in areas that have larger numbers of younger Black and other minority women. Finally, under the ACR demand specification, all five new mammography sites were proposed for the southern, more rural part of the state.

These results illustrate that decisions about allocating mammography screening resources are impacted by which set of screening guidelines are adopted. Adopting ACR guidelines, which recommend all women initiate annual screening mammography beginning at age 40, would lead to a greater focus on improving access in rural areas. The USPSTF guidelines would lead to a similar allocation, albeit with a small shift in allocation to more populous areas. Adopting the USPSTF guidelines inclusive of a race-based approach to screening would lead to a greater allocation of mammography resources to more populous and racially diverse geographic areas.

This study, which to our knowledge represents the first location-allocation analysis of geographic access to screening mammography under multiple screening guideline demand specifications, highlights the potential need to increase access to screening mammography for younger Black women and in rural areas. This study has several limitations. First, our analyses focused only on Delaware and findings may not apply to other states or geographic regions.^56^ Delaware has notably elevated rates of breast cancer among Black women under age 50,^43^ including rates of more aggressive subtypes of breast cancer,^44^ and therefore represents an important state in its own right to assess. Other states with similar profiles that may warrant similar assessments include those that overlap with the lower Mississippi Delta Region.^59^ Beyond racial disparities, this study did not examine mammography access for other high-risk groups (e.g., Ashkenazi Jewish women).^60^ Second, drive time represented our proxy for accessibility. For women accessing mammography facilities via other means (e.g., public transportation) and for whom other barriers limit access (e.g., hours of operation, insurance, childcare),^61^ our analysis may not fully capture these complex patterns. For example, while ownership of a vehicle was more limited in the urban areas of New Castle County, the number of bus stops was greater; one federally qualified health center in Wilmington previously noted that over 50% of their patients rely on busses for transit.^62^ Therefore, future studies of access should consider the time it would take to reach a mammography site via public transportation, as well as measures of other types of barriers, and mammography facility capacity. This research could inform the development of other interventions designed to close disparities in access to screening mammography, such as community outreach and transportation.

A third limitation of this study was the use of BICOE designation as a quality measure. Prior research found that breast cancer diagnoses made at BICOE-designated facilities are less likely to be a later stage,^37^ but it remains unclear what explains this association. BICOE designation requires ACR accreditation in mammography and stereotactic biopsy, breast ultrasound and ultrasound-guided biopsy, and breast MRI and MRI biopsy or the ability to refer the patient for MRI/MRI biopsy to another facility with a referral relationship. Therefore, an ACR accredited mammography unit at a BICOE facility is not necessarily of higher quality than an ACR accredited unit at a non-BICOE facility. It may not be necessary, let alone feasible, to convert a mammography facility to a BICOE to improve access to mammography. There is also not an established relationship between BICOE-designated facility and radiologist characteristics. Separate research reported a relationship between radiologist characteristics (i.e., qualifications, affiliation, and experience) and false-negative rates (i.e., missed breast cancer detection), particularly for racial/ethnic minorities and lower-income women.^63–65^

To conclude, drawing on the definition that health disparities represent potentially avoidable differences in disease outcomes,^66^ ensuring equitable geographic access to high-quality screening mammography facilities could help to close breast cancer disparities observed by race and rurality. However, making decisions about how to allocate mammography resources to ensure equitable access is contingent on which set of breast cancer screening guidelines are adopted, among other considerations (e.g., certificate of need). Given a set of guidelines, catchment and location-allocation analyses can guide the selection of locations for new mammography facilities and represent important methodological tools that can be leveraged in service of health equity. Future studies should collect additional data on access, quality, and capacity across geographic areas and population subgroups to facilitate the generation of more finely tuned and potentially impactful recommendations for the allocation of mammography facilities.

## Figures and Tables

**Table 1:** Census tract measures of population, transportation, and mammography sites in Delaware overall and by county.

	*Mean (standard deviation)*		
Measure	Delaware	New Castle County	Kent & Sussex Counties
N. tracts	214	129	85
Population	943,732	555,036	388,696
Deprivation^a^	0 (1)	0 (1)	0 (1)
N. women 40–49	277 (188)	284 (193)	266 (181)
N. women 50–74	719 (384)	649 (328)	824 (437)
N. women >74	176 (108)	155 (102)	209 (109)
Percent Black women	22 (22)	26 (25)	16 (14)
Percent w/ vehicles	93 (9)	91 (11)	95 (4)
N. bus stops	11 (10)	14 (11)	7 (8)
N. mammography sites	0.1 (0.4)	0.1 (0.4)	0.2 (0.5)
N. mammography units	0.2 (0.8)	0.2 (0.9)	0.2 (0.7)
N. BICOE sites	<0.1 (0.2)	0.1 (0.3)	< 0.1 (0.2)
N. BICOE units	0.1 (0.7)	0.1 (0.8)	0.1 (0.3)

BICOE, Breast Imaging Center of Excellence

a Operationalized as a Z-score composite of census tract indicators for education, employment, income and poverty, and household composition.

**Table 2:** Poisson regression models predicting the number of mammography facilities and units by census tract measures in Delaware and by county. Estimates may be interpreted as relative risks with corresponding 95% confidence intervals. Bold font denotes statistical significance.

	Delaware		New Castle County		Kent & Sussex Counties	
Measure	Number of facilities	Number of units	Number of facilities	Number of units	Number of facilities	Number of units
Deprivation^a^	0.98 (0.54, 1.67)	0.93 (0.56, 1.48)	0.97 (0.40, 2.13)	0.89 (0.42, 1.71)	0.58 (0.15, 2.04)	0.39 (0.12, 1.20)
N. women 40–49^b^	**1.89 (1.15, 3.24)**	**2.09 (1.33, 3.41)**	**3.03 (1.35, 7.23)**	**4.92 (2.33, 11.3)**	1.82 (0.89, 4.26)	1.88 (0.99, 4.04)
N. women 50–74^b^	0.77 (0.39, 1.44)	0.62 (0.34, 1.11)	0.42 (0.16, 1.09)	**0.26 (0.11, 0.59)**	0.89 (0.25, 2.64)	0.70 (0.23, 1.88)
N. women > 74^b^	1.09 (0.69, 1.65)	1.21 (0.83, 1.72)	1.33 (0.75, 2.25)	1.44 (0.90, 2.29)	1.00 (0.40, 2.17)	1.26 (0.61, 2.46)
Percent Black women^b^	**0.39 (0.17, 0.81)**	**0.36 (0.18, 0.66)**	**0.28 (0.07, 0.87)**	**0.16 (0.05, 0.49)**	0.48 (0.13, 1.33)	0.54 (0.20, 1.24)
Percent w/vehicles^b^	**0.41 (0.22, 0.80)**	**0.42 (0.24, 0.75)**	0.37 (0.11, 1.39)	0.37 (0.12, 1.44)	**0.46 (0.21, 0.94)**	**0.45 (0.24, 0.83)**
N. bus stops^b^	**1.55 (1.14, 2.07)**	**1.99 (1.59, 2.48)**	1.43 (0.92, 2.10)	**2.17 (1.61, 2.95)**	**1.95 (1.05, 3.82)**	**2.17 (1.28, 3.89)**

a Operationalized as a Z-score composite of census tract indicators for education, employment, income and poverty, and household composition.

b Centered and scaled for modeling.

## Data Availability

Datasets generated for this study are based on publicly available sources are available from the corresponding author on reasonable request.
